# Emergency admission and survival from aggressive non-Hodgkin lymphoma: A report from the UK's population-based Haematological Malignancy Research Network

**DOI:** 10.1016/j.ejca.2017.03.013

**Published:** 2017-06

**Authors:** Eleanor Kane, Debra Howell, Alexandra Smith, Simon Crouch, Cathy Burton, Eve Roman, Russell Patmore

**Affiliations:** a Epidemiology & Cancer Statistics Group, Department of Health Sciences, University of York, York, YO10 5DD, UK; b Haematological Malignancy Diagnostic Service, Bexley Wing, St James's University Hospital, Leeds, LS9 7TF, UK; c Queen's Centre for Oncology and Haematology, Castle Hill Hospital, Cottingham, HU16 5JQ, UK

**Keywords:** Non-Hodgkin lymphoma, Emergency presentation, Survival, Diffuse-large b-cell lymphoma

## Abstract

**Background:**

Non-Hodgkin lymphoma (NHL) is often diagnosed after emergency presentation, a route associated with poor survival and an indicator of diagnostic delay. Accounting for around half of all NHLs, diffuse large B-cell lymphoma (DLBCL) is of particular interest since although it is potentially curable with standardised chemotherapy it can be challenging to identify at an early stage in the primary care setting.

**Patients and methods:**

Set within a socio-demographically representative United Kingdom population of around 4 million people, data are from an established patient cohort. This report includes all patients (≥18 years) diagnosed with DLBCL 2004–2011 (n = 1660). Emergency admissions were identified via linkage to Hospital Episode Statistics using standard methods, and survival was examined using proportional hazards regression.

**Results:**

Two out of every five patients were diagnosed following an emergency admission, and this was associated with advanced disease and poor survival (p < 0.001). Among the 80% of patients treated with curative chemotherapy, survival discrepancies emerged at the point of diagnosis; the adjusted hazard ratio (emergency versus non-emergency) at one month being 4.0 (95% confidence interval 1.9–8.2). No lasting impact was evident in patients who survived for 12 months or more.

**Conclusion:**

Emergency presentation impacts negatively on DLBCL survival; patients presenting via this route have significantly poorer outcomes than patients with similar clinical characteristics who present via other routes.

## Highlights

• Emergency presentation has a marked impact on non-Hodgkin lymphoma (NHL) survival, not explained by disease stage or treatment.
• Emergency presentation among patients with diffuse large B-cell lymphoma is 50% higher in the United Kingdom than national data on non-Hodgkin lymphomas suggest.
• Survival benefits from earlier diagnosis of potentially curable NHLs could exceed that of new treatments.

## 1 Introduction

Cancer survival rates are reported to be poorer in Britain than many other European countries, resulting in an estimated 7000 avoidable premature deaths each year [1]. The evidence suggests that diagnostic delay is a major contributor to these differences, and hence the promotion of early diagnosis is being tackled through policy guidance and targets, with progress being audited nationally [2], [3], [4], [5], [6]. Nonetheless, despite some positive changes, there is considerable scope for improvement [7].

Emergency presentation is often considered a crude marker of diagnostic delay for cancers that commonly present with early signs and symptoms [8], [9]; the analysis of routinely compiled health data confirming that this route to diagnosis is associated with long intervals and poorer outcomes [10]. Among haematological cancers (lymphomas, myelomas and leukaemias), emergency presentation is relatively common [10], [11]. While this is clearly the appropriate route for conditions like the acute leukaemias, the reasons why a relatively large proportion of patients with non-Hodgkin lymphomas (NHL) present as an emergency and have poorer survival is less obvious.

As a group, NHLs are challenging to study since they comprise a heterogeneous spectrum of cancers with diverse patterns of onset, treatments and outcomes; the pathways of patients diagnosed with incurable but comparatively indolent subtypes, like follicular lymphoma and marginal zone lymphoma, tend to follow a remitting-relapsing course with periods of observation being interspersed with multiple lines of chemotherapy, whereas those of patients with more aggressive subtypes tend to dichotomise according to whether the cancer is potentially curable or not [12], [13]. In this context, diffuse large B-cell lymphoma (DLBCL), which is the commonest haematological malignancy and accounts for around half of all NHLs, is of particular interest since although it is curable with standardised chemotherapy administered over a 6–8 month period, patients who present with advanced disease tend to do less well than those diagnosed at an earlier stage [14], [15], [16], [17].

In the general patient population, DLBCL 5-year overall survival is now around 60%, disease/treatment-related deaths being highest in the first few months following diagnosis [14], [15], [16], [18]. Focussing on deaths occurring within 3 years of diagnosis, the present report uses data from an established United Kingdom (UK) patient cohort to examine the potential impact of emergency presentation on outcome in patients with DLBCL.

## 2 Methods

The study is set within the Haematological Malignancy Research Network (HMRN: www.hmrn.org), a population-based patient cohort instigated in 2004 to generate ‘real world’ evidence-based data for research and audit purposes [19]. HMRN's catchment population of around 4 million is socio-demographically similar to that of the UK as a whole [20]. Patient care within HMRN is provided by 14 hospitals, clinical practice adheres to national guidelines and all diagnoses (over 2200 new patients annually) are made and coded to the latest World Health Organisation (WHO) classification [12], [19], [21] by clinical specialists at a single integrated haematopathology laboratory (the Haematological Malignancy Diagnostic Service: www.hmds.info); which was cited in the UK's Cancer Reform Strategy as ‘the model for delivery of complex diagnostic services’ [2].

HMRN operates with Section 251 support under the National Health Service (NHS) Act 2006, and all patients have prognostic, full treatment and outcome data collected to clinical trial standards. All HMRN patients are ‘flagged’ for death at the national Medical Research Information Service and are routinely linked to Hospital Episode Statistics Admitted Patient Care (HES-APC) data. Area-based population counts are sourced from the Office for National Statistics; with the income domain of the national index of deprivation being used as a marker of socio-economic status [15], [22].

The present report focusses on patients aged 18 years or over who were newly diagnosed with *de novo* DLBCL between September 2004 and March 2011; all of whom were followed up for death for a minimum of 3 years. Primary source information on cancer stage, performance status, disease-associated systemic symptoms (B-symptoms), nodal status and treatment were obtained directly from medical records [15]. Following guidelines outlined by NHS Digital, hospital admissions were constructed from HES-APC. Using a similar approach to the Routes to Diagnosis initiative [10], emergency presentation was defined as an admission within 30 days of diagnosis directly from the accident and emergency (A&E) department (HES-APC admission method codes 21, 28), consultant-led outpatient clinic (code 24), bed bureau (code 23) or following a request from a General Practitioner (GP) (code 22).

All analyses were conducted using standard methods in the statistical packages Stata 14.1 (StataCorp, Texas) and R 3.2.2 (R Core Team, Vienna). Three-year survival was examined using time-to-event analysis. Adjusted survival curves were produced using the average approach; using Cox proportional hazards regression, survival curves were estimated for all possible combinations of covariate values and a weighted mean of the curves was calculated to adjust the mix among patients presenting as an emergency to that of those presenting via other routes [23]. The adjusted curve weights were propensity scores; logistic regression, adjusting for all covariates and their statistically significant interactions, was used to predict each patient's probability of presenting as an emergency, before scaling the predicted probabilities to the proportion of patients in each group.

## 3 Results

Of the 1660 patients diagnosed with DLBCL during the study period, 653 (39%) presented as an emergency. No statistically significant differences between those who presented via this route and those who did not were evident for gender, age at diagnosis and deprivation (Table 1). However, scores for patient's performance status and symptom burden (B-symptoms), cancer stage and nodal status, as well as the composite prognostic index, were all significantly higher in the group that presented as an emergency than in the group that did not (p < 0.001). Assignment of nodal status and cancer stage in DLBCL usually requires a bone marrow biopsy as well as a computed tomography (CT) and/or positron-emission tomography (PET) scan. In our data, 180 (11%) patients did not have enough information to assign nodal status and 165 (10%) could not be assigned a cancer stage, the slightly lower number with missing stage reflected the fact that a few of the 180 patients were assigned to stage IV on the basis of biopsy detected bone marrow, liver or lung involvement. For both parameters, lack of assignment occurred more frequently in patients who presented via the emergency route than among those presenting via the non-emergency route; 15% and 8%, respectively, for nodal status, and 13% and 8% for stage.

**Table 1:** Patient and disease characteristics distributed by presentation route: diffuse large B-cell lymphomas diagnosed Sept 2004 to Mar 2011 and followed for 3 years.

		Diagnoses by presentation route				Deaths by presentation route			Hazard ratio (95% confidence interval)^b^
				Diagnoses (%)	Non-emergency (%)	Emergency (%)	Chi-square^b^	Deaths (% of all diagnoses)		Non-emergency (% of non-emergency presentations)	Emergency (% of emergency presentations)
	Total	1660 (100)	1007 (100)	653 (100)		749 (45)	346 (34)	403 (62)	2.5 (2.2–2.9)
Sex	Male	853 (51)	529 (53)	324 (50)		380 (45)	181 (34)	199 (61)	2.6 (2.1–3.2)
	Female	807 (49)	478 (47)	329 (50)	p = 0.25	369 (46)	165 (35)	204 (62)	2.4 (2.0–3.0)
*Age at diagnosis (years)*	<50	214 (13)	125 (12)	89 (14)		39 (18)	10 (8)	29 (33)	4.8 (2.4–9.9)
	50–74	873 (53)	538 (53)	335 (51)		345 (40)	155 (29)	190 (57)	2.7 (2.2–3.3)
	75+	573 (35)	344 (34)	229 (35)	p = 0.64	365 (64)	181 (53)	184 (80)	2.6 (2.1–3.2)
	Median (IQR)	*69.9 (59.2–78.1)*	*69.2 (59.2–77.9)*	*70.6 (58.9–78.4)*		*74.8 (65.6–81.6)*	*75.6 (65.8–82.1)*	*74.3 (65.1–81.4)*	
*Deprivation (quintile)*^a^	1, Least deprived	352 (21)	234 (23)	118 (18)		150 (43)	79 (34)	71 (60)	2.5 (1.8–3.5)
	2	386 (23)	221 (22)	165 (25)		163 (42)	65 (29)	98 (59)	2.8 (2.0–3.8)
	3	326 (20)	205 (20)	121 (19)		158 (48)	81 (40)	77 (64)	2.3 (1.7–3.2)
	4	292 (18)	175 (17)	117 (18)		140 (48)	63 (36)	77 (66)	2.6 (1.8–3.6)
	5, Most deprived	302 (18)	171 (17)	131 (20)	p = 0.08	136 (45)	57 (33)	79 (60)	2.5 (1.8–3.5)
*Patient's performance status*^a^	0, Good	430 (26)	364 (36)	66 (10)		79 (18)	60 (16)	19 (29)	1.9 (1.2–3.2)
	1	719 (43)	467 (46)	252 (39)		287 (40)	164 (35)	123 (49)	1.7 (1.3–2.1)
	2	335 (20)	133 (13)	202 (31)		230 (69)	88 (66)	142 (70)	1.3 (1.0–1.7)
	3 + 4 Poor	152 (9)	33 (3)	119 (18)	p < 0.001	137 (90)	28 (85)	109 (92)	1.5 (1.0–2.3)
*B-symptoms*	Absent	919 (55)	610 (61)	309 (47)		368 (40)	180 (30)	188 (61)	3.0 (2.4–3.6)
	Present	741 (45)	397 (39)	344 (53)	p < 0.001	381 (51)	166 (42)	215 (62)	2.0 (1.7–2.5)
*Nodal status*	Nodal	441 (30)	325 (35)	116 (21)		125 (28)	77 (24)	48 (41)	2.1 (1.4–3.0)
	Extranodal	281 (19)	163 (18)	118 (21)		112 (40)	52 (32)	60 (51)	1.9 (1.3–2.8)
	Nodal + extranodal	758 (51)	438 (47)	320 (58)	p < 0.001	379 (50)	170 (39)	209 (65)	2.5 (2.0–3.0)
	Not assigned	180	81	99		133 (74)	47 (58)	86 (87)	2.7 (1.9–3.8)
*Cancer stage*	I	258 (17)	200 (22)	58 (10)		51 (20)	35 (18)	16 (28)	1.7 (0.9–3.1)
	II	302 (20)	216 (23)	86 (15)		86 (28)	47 (22)	39 (45)	2.6 (1.7–4.0)
	III	233 (16)	169 (18)	64 (11)		88 (38)	55 (33)	33 (52)	2.1 (1.3–3.2)
	IV	702 (47)	342 (37)	360 (63)	p < 0.001	406 (58)	163 (48)	243 (68)	2.0 (1.6–2.4)
	Not fully staged	165	80	85		118 (72)	46 (58)	72 (85)	2.4 (1.7–3.5)
*International Prognostic Index (IPI)*	Low	347 (26)	273 (33)	74 (15)		52 (15)	40 (15)	12 (16)	1.1 (0.6–2.1)
	Low/intermediate	303 (23)	216 (26)	87 (18)		83 (27)	46 (21)	37 (43)	2.4 (1.5–3.7)
	Intermediate/high	318 (24)	200 (24)	118 (24)		135 (42)	78 (39)	57 (48)	1.4 (1.0–2.0)
	High	349 (26)	145 (17)	204 (42)	p < 0.001	247 (71)	89 (61)	158 (77)	1.8 (1.4–2.4)
	Not known	343	173	170		232 (68)	93 (54)	139 (82)	2.6 (2.0–3.3)
*1st line chemotherapy with curative intent*	Yes	1346 (81)	883 (88)	463 (71)		468 (35)	249 (28)	219 (47)	2.1 (1.7–2.5)
	No	314 (19)	124 (12)	190 (29)	p < 0.001	281 (89)	97 (78)	184 (97)	2.5 (1.9–3.2)
	*Palliative/supportive*	*217 (13)*	*67 (7)*	*150 (23)*		*217 (100)*	*67 (100)*	*150 (100)*	*2.1 (1.6–2.9)*
	*Localised disease*	*97 (6)*	*57 (6)*	*40 (6)*	*p < 0.001*	*64 (66)*	*30 (53)*	*34 (85)*	*2.5 (1.5–4.0)*

a Not known: deprivation (n = 2), performance status (n = 24).

b Hazard ratios were estimated using Cox regression.

The pronounced tendency for patients presenting via the emergency route to have more advanced disease impacted on whether or not they were fit enough to be treated with intensive chemotherapy with curative intent; 71% (n = 463) of those presenting as an emergency receiving such treatment, compared to 88% (n = 883) of those presenting via other routes (p < 0.001). Of the 1346 patients treated with potentially curative chemotherapy, 85% received standard R-CHOP (rituximab + cyclophosphamide, doxorubicin, vincristine and prednisone), and the remainder were mainly treated with R-CVP (rituximab + cyclophosphamide, vincristine and prednisone) or R-CODOX-M/R-IVAC (rituximab + vincristine, doxorubicin, cyclophosphamide, cytarabine, etoposide, ifosfamide, mesna and methotrexate) [17]; no regimen differences were evident by route of presentation. The 314 (19%) of patients who were not treated intensively comprised a heterogeneous group; most (n = 217/314; 69%) were managed using a palliative/supportive approach, the remaining 97 had localised disease that was mainly treated with radiotherapy. The proportion of patients receiving palliative/supportive care was significantly higher among patients presenting via the emergency route (Table 1).

**Fig. 1:**
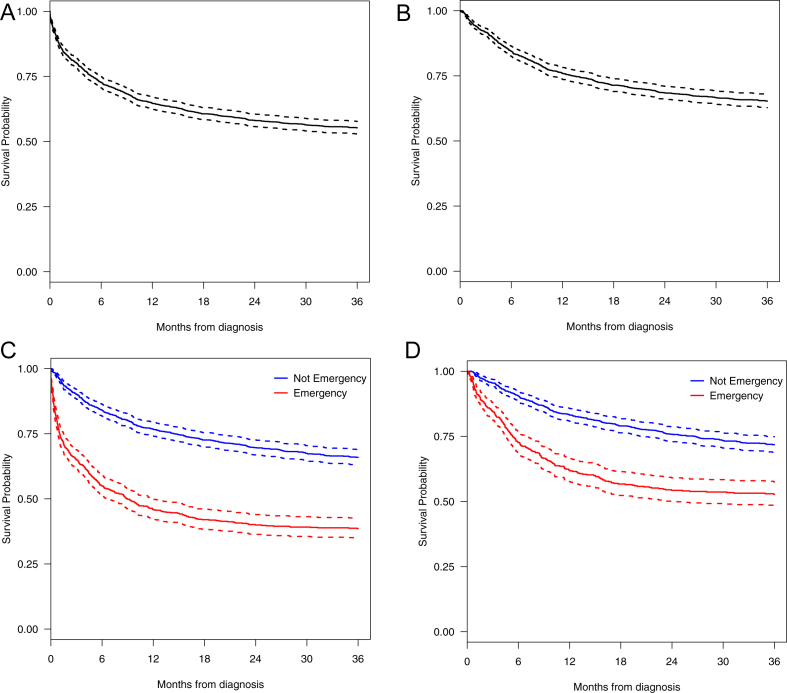
Kaplan–Meier survival curves and 95% confidence intervals (dotted lines) for patients with diffuse large B-cell lymphoma: total patients (A), patients treated with curative intent (B), total patients stratified by mode of presentation (C) and curatively treated patients stratified by mode of presentation (D).

During the 3 years following diagnosis, 749 (45%) of the patients died. As might be expected, the cumulative incidence (risk) of death among patients who presented via the emergency route was almost twice (403/653, 62%) that of those who presented via other routes (346/1007; 34%). This survival difference, which is evident within all strata of Table 1, emerged at the point of diagnosis (Fig. 1) and yielded an overall unadjusted hazard ratio (HR) for the 3 year period of 2.5 (95% confidence interval [CI] 2.2–2.9) (Table 1). Interestingly, within age strata the strongest effect was seen among those who were diagnosed before the age of 50 years (HR 4.8, 95% CI 2.4–9.9).

The survival disparity between those who presented via the emergency route and those who did not is as evident among patients who were treated with intensive chemotherapy as it is among all patients combined (Fig. 1C and 1D). Table 2 presents conditional and cumulative survival estimates (adjusted for cancer stage and nodal involvement, patient's age, deprivation, performance status and B-symptoms) distributed by time since diagnosis; the 1-month (30 day) HRs for all patients (n = 1660; HR = 3.9, 95% CI 2.7–5.6), those treated with intensive chemotherapy (n = 1346; HR = 4.0, 95% CI 1.9–8.2), and those who were managed with supportive/palliative care (n = 217; HR = 3.8, 95% CI 2.3–6.1) are broadly similar. Among the 80% of patients who received intensive chemotherapy, this survival separation is retained throughout the treatment period; the conditional HR estimates show progressively smaller differences reaching 1.3 (95% CI 0.9–1.9) during the 6–12 month period. Importantly, however, mode of presentation had no lasting impact among patients who responded to chemotherapy and survived for 12 months or more; the conditional 12–24 year HRs being 1.0 (95% CI 0.7–1.5) for those treated with curative intent (Table 2).

**Table 2:** Overall and conditional hazard ratios (HR) and 95% confidence intervals (95% CI) distributed by treatment and mode of presentation.

Time since diagnosis (months)		All patients					1st line chemotherapy with curative intent					Palliative/supportive care				
		Non-emergency		Emergency		Hazard ratio^a^ (95% CI)	Non-emergency		Emergency		Hazard ratio^a^ (95% CI)	Non-emergency		Emergency		Hazard ratio^a^ (95% CI)
		Patients	Deaths	Patients	Deaths		Patients	Deaths	Patients	Deaths		Patients	Deaths	Patients	Deaths	
Conditional	0–1	1007	41	653	164	3.9 (2.7–5.6)	883	10	463	34	4.0 (1.9–8.2)	67	28	150	126	3.8 (2.3–6.1)
Survival	1–3	966	61	489	67	1.5 (1.1–2.2)	873	29	429	37	1.8 (1.1–3.0)	39	27	24	16	1.4 (0.7–2.8)
	3–6	905	62	422	65	1.5 (1.1–2.2)	844	44	392	55	1.9 (1.3–2.8)	12	7	8	6	1.4 (0.4–4.3)
	6–12	843	74	357	60	1.3 (0.9–1.9)	800	65	337	51	1.3 (0.9–1.9)	5	4	2	2	(Not estimated)
	12–24	769	71	297	38	1.0 (0.7–1.5)	735	66	286	35	1.0 (0.7–1.5)	1	1	0	0	(Not estimated)
	24–36	698	37	259	9	0.5 (0.2–0.9)	669	35	251	7	0.4 (0.2–0.8)	0	0	0	0	(Not estimated)
Cumulative	0–1	1007	41	653	164	3.9 (2.7–5.6)	883	10	463	34	4.0 (1.9–8.2)	67	28	150	126	3.8 (2.3–6.1)
Survival	0–3	1007	102	653	231	2.5 (1.9–3.2)	883	39	463	71	2.4 (1.6–3.6)	67	55	150	142	2.8 (1.9–4.2)
	0–6	1007	164	653	296	2.1 (1.7–2.6)	883	83	463	126	2.1 (1.6–2.9)	67	62	150	148	2.7 (1.8–3.9)
	0–12	1007	238	653	356	1.9 (1.6–2.3)	883	148	463	177	1.8 (1.4–2.3)	67	66	150	150	2.6 (1.8–3.8)
	0–24	1007	309	653	394	1.7 (1.5–2.0)	883	214	463	212	1.6 (1.3–1.9)	67	67	150	150	2.6 (1.8–3.8)
	0–36	1007	346	653	403	1.6 (1.4–1.9)	883	249	463	219	1.4 (1.1–1.7)	67	67	150	150	2.6 (1.8–3.8)

a Hazard rate ratios were estimated using Cox regression adjusting for age, deprivation, performance status, B symptoms, cancer stage and nodal involvement.

**Fig. 2:**
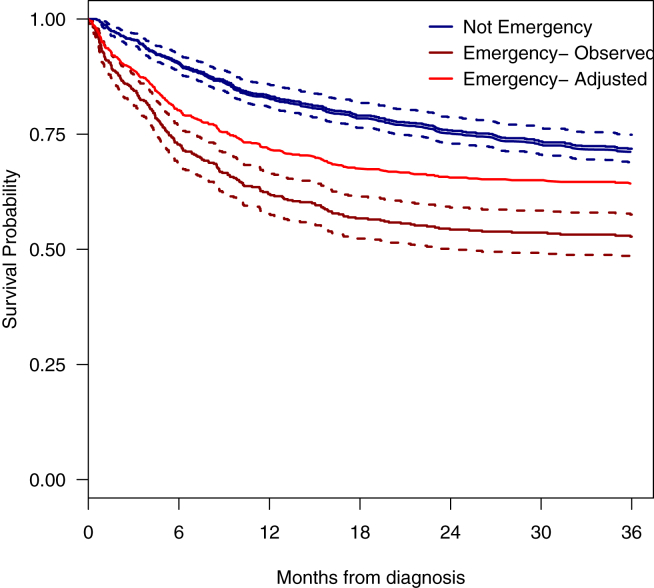
Average adjusted survival curve among patients treated with curative intent. Survival curves were predicted from Cox proportional hazards model adjusted for age, deprivation, performance status, B symptoms and disease stage, and the average adjusted curve is shown with the observed Kaplan–Meier survival curves for emergency and not emergency presentation.

The contribution of potentially confounding prognostic factors (cancer stage, nodal status, age, performance status, B-symptoms and deprivation) to the survival dichotomy seen even among patients treated curatively who present via different routes is demonstrated in Fig. 2. Clearly, whilst such established risk factors explain some of the variation seen in the initial months following diagnosis, they do not account for it all.

## 4 Discussion

Survival of patients diagnosed with DLBCL who presented via the emergency route was significantly worse than that of patients who presented via other routes. Whilst emergency presentation is the appropriate route for several conditions and illnesses, including the acute leukaemias, for the majority of cancers it is considered to be a proxy for advanced disease and delayed diagnosis [8], [9]. Two out of 5 patients in our population-based cohort were diagnosed via this route and the survival disparity, which emerged at the point of diagnosis, was not wholly explained by stage of disease, performance status, symptom burden and area-based deprivation. In this context, although our findings for DLBCL confirm the association between emergency presentation, advanced disease and outcome, they also suggest that other unmeasured factors may have a role to play. Importantly, however, the survival difference, which was as striking among the 80% of patients who were treated with intensive chemotherapy as among the total patient group, did not extend beyond the first year.

This is the first population-based study to examine the potential impact of emergency presentation on survival among an unselected group of patients diagnosed with DLBCL, the commonest of the NHLs. The paucity of ‘real-world’ information on clinically meaningful NHL subtypes is primarily due to the complexity of disease classification [12], [24]; which means that national data are often reported for all NHL subtypes combined. In England, for example, the Routes to Diagnosis initiative found that around one in four NHLs were diagnosed via the emergency route, which is fewer than the two in five observed here for DLBCL [10]. This difference is likely to reflect the heterogeneity of NHLs which, in addition to aggressively presenting cancers like DLBCL, Burkitt lymphoma and mantle cell lymphoma, include incurable but comparatively indolent subtypes such as follicular lymphoma and marginal zone lymphoma, which tend to present less acutely [13].

That advanced disease is a poor prognostic factor for most cancers, including DLBCL [16], is well established; and as such, earlier diagnosis has been identified as key to improving cancer outcomes in the UK [6], [25]. In this context, the National Cancer Patient Experience Survey reported that around a third of patients with NHL (aggressive and indolent subtypes combined) had three or more pre-referral GP consultations; and many patients have expressed general frustrations with the diagnostic process [4], [26], [27], [28]. Such difficulties are generally attributed to the symptoms of lymphoma, which can be vague, intermittent and frequently associated with self-limiting conditions [29], [30], [31]. To our knowledge, only one small study (n = 278) has attempted to examine diagnostic delay in the primary care setting, and no impact on survival was detected in patients diagnosed during 2002–2010 [30]. However, mode of presentation was not examined and the study was restricted to patients who were treated with intensive chemotherapy; interestingly, patients were also younger and had less advanced disease (63 years, 50% with a performance status of zero) than similarly treated patients in our cohort (67.4 years, 32% with a performance status of zero) [15].

Major strengths of our study include its large well-defined catchment population, completeness of ascertainment and world-class diagnostics. All lymphomas within the study area's 14 hospitals were diagnosed and coded to the latest WHO oncology classification at one of the largest integrated haematopathology laboratories in Europe, which is regarded by the UK's National Institute for Health and Care Excellence as the model for service delivery [2], [3]. In addition, unlike analyses based solely on administrative databases, we were able to incorporate information on key clinical parameters, including patient's performance status and cancer stage, into our investigations; enabling us to show that patients presenting as an emergency had poorer survival than those with similar clinical characteristics who presented via other routes.

With respect to potential limitations, emergency presentation is a proxy for what is likely to be a complex underpinning set of events/variables, some of which will be related to the cancer itself and some of which will not. In this context, whilst socio-economic patterning is frequently detected in cancers with strong environmental/life-style risk factors and/or screening programmes, relationships with haematological cancers are less clear-cut. Within our UK population of 4 million, no socio-economic associations with incidence for any haematological malignancy subtype have been observed [20]; and although variations with area-based measure of deprivation and survival have been detected for chronic myeloid leukaemia (a long-term condition controlled with daily oral therapy) [33], no evidence of socio-economic patterning for DLBCL incidence or outcome have been found [15]. Nonetheless other factors, such as travelling distance to hospital and the presence of existing co-morbidities, may well have had an important role to play in the findings presented in this report [34]. Furthermore, it is important to note that, in common with the national Routes to Diagnosis study [10], [32], emergency admissions from several sources have been grouped together. In our data, around 30% of emergency admissions to hospital were directly requested by GPs, and a further 40% occurred via A&E department; the latter comprising a complex mix of self-presentations, as well as A&E referrals initiated by GPs. In this context, the ability to link to primary care databases would enable more effective pathway mapping. Such routine linkage, which should be possible in the future, could lead to increased understanding and better strategies to support earlier cancer diagnosis.

In summary, patients diagnosed with DLBCL after an emergency admission had poorer levels of fitness, more advanced stage disease, and were less likely to be treated with curative intent. Among the 80% of patients treated curatively, the survival of those presenting via an emergency route was significantly poorer than that of patients with similar clinical characteristics who presented via other routes. This survival disparity emerged at the point of diagnosis, but did not extend beyond the first year. Given the curable nature of this cancer, strategies to support earlier diagnosis should be considered, since even minor improvements in time to diagnosis could lead to significant survival benefits in the longer term.

## Contributors

DH, RP, ER and AS had the idea for the study. EK carried out the analysis in collaboration with SC, AS and ER; DH, EK and ER drafted the manuscript and RP and CB commented on the clinical aspects. All authors commented on and approved the final draft.

## Conflict of interest statement

None declared.

## Funding

This study used data from the Haematological Malignancy Research Network (www.hmrn.org), which was funded by Bloodwise (formerly Leukaemia & Lymphoma Research), and received support from the NHS clinical and administrative staff across the 14 hospitals in the study area.

## Ethics statement

The Haematological Malignancy Research Network (HMRN) has ethical approval (REC 04/01205/69) from Leeds West Research Ethics Committee, R&D approval from each Trust, and exemption from Section 251 (formally Section 60) of the Health and Social Care Act (2001) (PIAG 1-05(h)/2007). Hospital Episode Statistics—Admitted Patient Care data for 2004–2011 are reused with permission from NHS Digital (formerly the Health and Social Care Information Centre).
